# The Disease Model of Addiction: The Impact of Genetic Variability in the Oxidative Stress and Inflammation Pathways on Alcohol Dependance and Comorbid Psychosymptomatology

**DOI:** 10.3390/antiox13010020

**Published:** 2023-12-21

**Authors:** Evangelia Eirini Tsermpini, Katja Goričar, Blanka Kores Plesničar, Anja Plemenitaš Ilješ, Vita Dolžan

**Affiliations:** 1 Pharmacogenetics Laboratory, Institute of Biochemistry and Molecular Genetics, Faculty of Medicine, University of Ljubljana, 1000 Ljubljana, Slovenia; evangelia.tsermpini@dal.ca (E.E.T.); katja.goricar@mf.uni-lj.si (K.G.); 2 University Psychiatric Clinic, 1000 Ljubljana, Slovenia; blanka.kores@psih-klinika.si; 3 Faculty of Medicine, University of Ljubljana, 1000 Ljubljana, Slovenia; 4 Department of Psychiatry, University Clinical Centre Maribor, 2000 Maribor, Slovenia

**Keywords:** alcohol addiction, alcohol-related psychosymptomatology, oxidative stress, inflammation, polymorphisms

## Abstract

Oxidative stress and neuroinflammation are involved in the pathogenesis of alcohol addiction. However, little is known regarding the effect of genetic, behavioral, psychological, and environmental sources of origin on the inflammation and oxidative stress pathways of patients with alcohol addiction. Our study aimed to evaluate the impact of selected common functional single-nucleotide polymorphisms in inflammation and oxidative stress genes on alcohol addiction, and common comorbid psychosymptomatology. Our study included 89 hospitalized alcohol-addicted patients and 93 healthy individuals, all Slovenian males. Their DNA was isolated from peripheral blood and patients were genotyped for *PON1* rs705379, rs705381, rs854560, and rs662, *SOD2* rs4880, *GPX1* rs1050450, *IL1B* rs1143623, rs16944, and rs1071676, *IL6* rs1800795, *IL6R* rs2228145, and miR146a rs2910164. Kruskal–Wallis and Mann–Whitney tests were used for the additive and dominant genetic models, respectively. Our findings suggested the involvement of *IL6* rs1800795 in alcohol addiction. Moreover, our data indicated that the genetic variability of *SOD2* and *PON1*, as well as *IL1B* and *IL6R,* may be related to comorbid psychosymptomatology, revealing a potential indirect means of association of both the oxidative stress and inflammation pathways.

## 1. Introduction

Alcohol addiction is a complex mental disorder characterized by an impaired control of alcohol use and it has a negative impact on overall health, disability, and death rates, worldwide [1]. 

Both genetic and environmental factors, such as age, sex, and socioeconomic variables, can influence alcohol consumption, possibly with combined effects [2]. A meta-analysis on heritability provided evidence that genetics is a crucial factor for alcohol use disorder, as it can be up to 50% heritable [3]. A recent study that included Europeans and Africans indicated that the genetic risk of alcohol abuse is transmitted from parents to offspring due to the influence of the home environment [4]. Other environmental and developmental risk factors include prenatal alcohol exposure and adverse childhood events, like abuse, neglect, or family dysfunction, although with modest effects. Moreover, drinking during the age of 18–25 years old, can potentially predict alcohol addiction in adulthood, which can lead to an impairment of psychosocial end points [2].

It is well-established that oxidative stress and neuroinflammation are involved in alcohol addiction. Preclinical and clinical studies have shown that alcohol metabolism is associated with oxidative stress. The low antioxidant activity of superoxide dismutase (SOD), catalase, and glutathione peroxidase (GPX) can lead to increased oxidative stress in alcohol addicted-patients, as well as in patients during detoxification treatment [5]. Paraoxonase-1 (PON1) is another antioxidant enzyme, which can prevent low-density lipoprotein oxidation and hydrolyses various substrates, including peroxides, thus acting as a defense against not only oxidative stress but also inflammation. PON1 also has an anti-inflammatory role, as it can also directly suppress macrophage pro-inflammatory responses [6]. 

Alcohol addiction can also be considered an inflammatory condition mostly due to the altered cytokine regulation found in many of the patients’ organs, including the brain [7]. Alcohol addiction can lead to endotoxemia and the increased secretion of pro-inflammatory cytokines, like interleukin-1β (IL-1β) and interleukin-6 (IL-6), which can alter various signaling pathways and cause damage in the central nervous system and other organs [8]. Furthermore, the gene expression levels of microRNAs, known for their involvement in the inflammatory response, such as miR-146a [9], were reduced in the peripheral blood mononuclear cells from patients with cirrhosis, and in extracellular vesicles from alcohol-drinkers without liver injury, when compared with non-drinkers [10].

However, to our knowledge, there are no studies that focus on the genetic variability of *PON1*, *SOD2*, *GPX1*, *IL1B*, *IL6*, interleukin 6 receptor (*IL6R*), and miR146a related to the risk of alcohol addiction, and patients’ comorbid psychosymptomatology. Aiming to fill this gap, we used genetic models to address the potential relationship between genetic factors and behavioral, psychological, and environmental factors in patients with alcohol addiction. More specifically, the present study investigated common functional genetic variations in the oxidative stress (*PON1*, *SOD2*, and *GPX1*) and inflammation (*IL1B*, *IL6*, *IL6R*, and miR146a) pathways and their potential association with alcohol addiction and alcohol-related comorbid psychosymptomatology, including obsessive–compulsive, social anxiety, depressive, anxious, and aggressive symptoms. 

## 2. Materials and Methods

### 2.1. Study Population 

This study was focused on two groups of participants: hospitalized patients receiving treatment for alcohol addiction, and healthy individuals with no alcohol addiction history. All enrolled participants were Slovenian males aged 18 to 66. Hospitalized alcohol-addicted patients were recruited at the University Clinical Centre, Maribor, and the University Psychiatric Clinic, Ljubljana. Their diagnosis was made by experienced psychiatrists, according to the DSM-IV [11] criteria of alcohol addiction, and patients presented no significant abstinence symptoms after hospitalization for at least two weeks. Alcohol-addicted patients who had a present or past diagnosis of dependence or abuse of other substances (except nicotine), bipolar disorder, major depression, schizophrenia, schizoaffective disorder, according to the DSM-IV, organic mental syndromes, head trauma, and neurological disorders, or other significant medical conditions potentially affecting the central nervous system, were excluded from the study. Healthy individuals were blood donors, matched for ethnicity and sex, with no DSM-IV axis I mental disorders, including alcohol addiction and a family history of alcohol dependence or other psychiatric disorders, as it was determined by the psychiatrists after a short clinical interview. 

This study was approved by the Slovenian National Medical Ethics Committee (approval No. 117/06/10 and 148/02/1011), and all participants agreed and signed informed consent forms, following the Declaration of Helsinki, after being informed about the aims of the study. 

Patients’ demographic data, including age, residence, marital status, academic years, and smoking status, along with their clinical data, were recorded at baseline. All participants completed questionnaires to evaluate their drinking habits, the severity of alcohol use and addiction, as well as comorbid symptomatology, including depression and anxiety symptoms, social anxiety symptoms, obsessive–compulsive traits, and symptoms of aggression. 

The Alcohol Use Disorders Identification Test (AUDIT) is a screening tool to identify hazardous drinkers or patients with alcohol abuse or addiction. It is a 10-item questionnaire that focuses on alcohol consumption, drinking behavior, and alcohol-related problems, and the higher the score, the more likely it is that the drinking is harmful and leads to alcohol addiction. The Obsessive Compulsive Drinking Scale (OCDS) is a scale that measures an individual’s alcohol use and his attempts to control his drinking. On the other hand, the Zung depression scale is a screening tool to identify the presence of depressive symptoms, ranging from mild to moderate and severe depression. Similarly, the Zung anxiety scale measures the anxiety levels of individuals who have anxiety-related symptoms. The Brief Social Phobia Scale (BSPS) is designed to assess the characteristic symptoms of social phobia, evaluating commonly feared or avoided situations of autonomic distress. The Yale–Brown Obsessive Compulsive Scale (YBOCS) obsession scale screens for unwanted thoughts, the fear of losing important things, concerns with order, symmetry, or exactness that intrude on thinking against a person’s wishes and efforts to resist them and usually involve themes of harm, risk, and danger. YBOCS compulsions are an assessment tool that can determine the severity of compulsions, i.e., repetitive, purposeful, intentional behaviors called rituals which urge people to do something to lessen feelings of anxiety or other discomfort. Finally, the Buss-Durkee Hostility Inventory (BDHI) is a screening test for measuring a person’s level of hostility, by assessing verbal and physical aggression, and anger. More information about the scales can be found in our previous articles [12,13,14].

### 2.2. Molecular Genetic Analysis 

DNA was extracted from whole blood using the QIAamp Blood Mini kit, according to the manufacturer’s protocols (Qiagen GmbH, Hilden, Germany). Genotyping of all SNPs was performed with competitive allele-specific PCR (KASP), using the KASP Master mix and custom KASP genotyping assays (LGC, Teddington, UK) according to the manufacturer’s instructions (KBiosciences, Herts, UK, and LGC Genomics, Teddington, UK). Thermal cycling conditions are presented in Supplementary Tables S16–S18.

### 2.3. Statistical Analysis 

The statistical analysis was carried out using the 27.0 version of IBM SPSS Statistics (IBM Corporation, Armonk, NY, USA). The cut-off for the level of significance was set at 0.05. The deviation from Hardy–Weinberg equilibrium (HWE) in healthy donors and for all studied polymorphisms was evaluated using Pearson’s chi-square test. Both additive and dominant genetic models were used for the analyses. In comparing the clinical characteristics of alcohol-addicted patients, Fisher’s exact test was used for the distribution of categorical variables, while the nonparametric Mann–Whitney test was used for the continuous ones. The association of polymorphisms with alcohol addiction was evaluated using logistic regression analysis, and odds ratios (ORs) and 95% confidence intervals (CIs) were determined. In the multivariable logistic regression, age, residence place, marital status, academic years, and smoking status were considered covariates, and significant variables were used for adjustment in regression analysis. Finally, Kruskal–Wallis and Mann–Whitney non-parametric tests for additive and dominant genetic models were used to associate genotypes with psychosymptomatology scores.

## 3. Results

Regarding the demographic characteristics of our cohort, the median age of the hospitalized alcohol-addicted patients was significantly higher than the healthy controls (*p* < 0.001), while more controls were smokers (*p* < 0.001) and had a partner (*p* = 0.002) (Table 1).

Regarding the questionnaires used to evaluate psychosymptomatology, we observed statistically significant differences between the patients and controls in all questionnaires’ scores, except the BSPS (*p* = 0.063) (Supplementary Table S1). 

The genotypes of the controls were all in HWE for all studied polymorphisms (all *p* > 0.05) (Supplementary Table S2).

After a comparison of the genotype frequencies between patients and controls, a statistically significant difference was observed for *IL6* rs1800795 CC (*p* = 0.038), which remained significant after adjustment for age, education, smoking, environment, and partnership (*p* = 0.043) (Table 2). No statistically significant differences were observed for the rest of the studied polymorphisms (Supplementary Table S3). Table 2 shows the comparison of the genotype frequencies of genetic variants of the inflammation pathway between hospitalized alcohol-addicted patients and healthy controls, and Supplementary Table S3 shows the comparison of genotype frequencies of genetic variants of the oxidative stress pathway between hospitalized alcohol-addicted patients and healthy controls.

**Figure 1:**
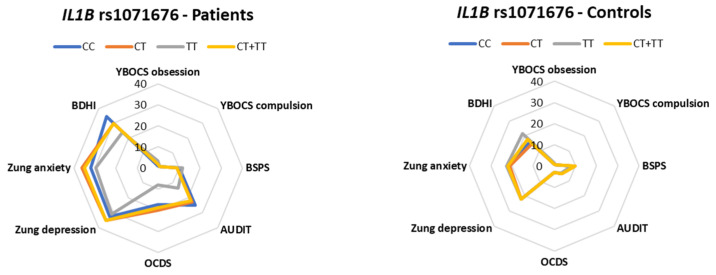

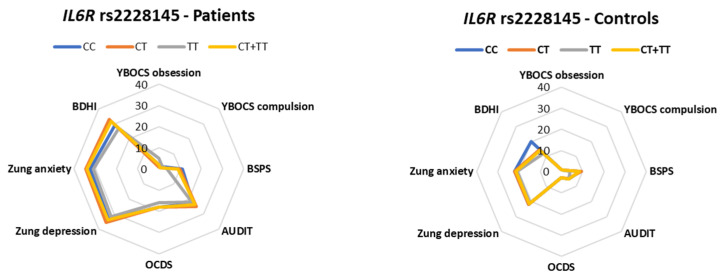
Polar diagrams that show the genetic variants of inflammation pathways that were statistically significant in at least one psychosymptomatology scale and in at least one group of either hospitalized alcohol-addicted patients or healthy individuals.

**Figure 2:**
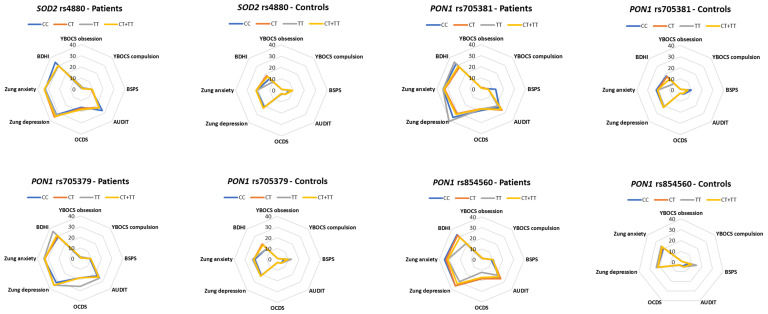
Polar diagrams that show the genetic variants of the oxidative stress pathways that were statistically significant in at least one psychosymptomatology scale and in at least one group of either hospitalized alcohol-addicted patients or healthy individuals.

Regarding potential associations between the studied polymorphisms and the scores of the comorbid psychosymptomatology scales, we found that *SOD2* rs4880 CT + TT genotypes were associated with a higher YBOCS obsessions subtotal (*p* = 0.016) and YBOCS compulsions subtotal (*p* = 0.046) in alcohol-addicted patients. Similarly, *PON1* rs705381 CT + TT was associated with lower a YBOCS compulsions subtotal in healthy controls (*p* = 0.027) and with a lower BSPS in alcohol-addicted patients (*p* = 0.041). *PON1* rs705379 GG was associated with BSPS (*p* = 0.001), Zung depression (*p* = 0.005), Zung anxiety (*p* = 0.002), and BDHI scores (*p* = 0.040) in healthy controls. Associations were also found with *PON1* rs705379 GA + AA in healthy individuals, i.e., BSPS (*p* = 0.002), Zung depression (*p* = 0.001), Zung anxiety (*p* = 0.001), and BDHI scores (*p* = 0.047). *PON1* rs854560 AA was associated with a YBOCS obsessions subtotal (*p* = 0.038), BSPS (*p* = 0.018), and Zung anxiety (*p* = 0.005). The YBOCS obsession subtotal and BSPS scores were also associated with *PON1* rs854560 AT + TT (*p* = 0.014 and *p* = 0.003, respectively). *IL1B* rs1071676 GG was associated with AUDIT scores in alcohol-addicted patients (*p* = 0.045). *IL6R* rs2228145 AA was associated with a YBOCs compulsions subtotal in alcohol-addicted patients (*p* = 0.033), whereas in healthy individuals, *IL6R* rs2228145 AA and AC + CC were both associated with BDHI scores (*p* = 0.014 and *p* = 0.004, respectively) (Figure 1 and Figure 2, Table 3 and Table 4). No statistically significant associations were observed between the rest of the studied polymorphisms and the psychosymptomatology scores either in the hospitalized alcohol-addicted patients or the healthy individuals (Supplementary Tables S4–S15).

## 4. Discussion

In this pilot study, we used genetic models to investigate the relationship between the genetic variability in oxidative stress- and inflammation-related pathways and the behavioral, psychological, and environmental factors for patients with alcohol addiction. Thus, we focused on the role of *PON1* rs705379, rs705381, rs854560, and rs662, *SOD2* rs4880, *GPX1* rs1050450, *IL1B* rs1143623, rs16944, and rs1071676, *IL6* rs1800795, *IL6R* rs2228145, and miR146a rs2910164 in alcohol addiction, and patients’ comorbid psychosymptomatology. 

We found a statistically significant association of *IL6* rs1800795 with alcohol addiction after a comparison of the genotype frequencies of alcohol-addicted patients and healthy individuals, which remained significant after adjustments for other parameters associated with alcohol addiction. No statistically significant associations with alcohol addiction were found for *PON1* rs705379, rs705381, rs854560, or rs662, *SOD2* rs4880, *GPX1* rs1050450, *IL1B* rs1143623, rs16944, or rs1071676, *IL6R* rs2228145, or miR146a rs2910164. We also observed statistically significant associations between *SOD2* rs4880 and obsessive–compulsive symptoms, *PON1* rs705381 and social phobia, *IL1B* rs1071676 and AUDIT scores, and *IL6R* rs2228145 and compulsions in alcohol-addicted patients. In the controls, *PON1* rs705381 was associated with compulsions, *PON1* rs705379 with social anxiety, depression, anxiety, and aggression, *PON1* rs854560 with obsessions, anxiety, and social phobia, and *IL6R* rs2228145 with aggression. 

Cytokines are important markers of systemic inflammation that can induce neuroinflammation and further affect mood, cognition, and drinking habits [15]. Many of them, including IL-6, have demonstrated neurodegenerative and neuroprotective dynamics and have been associated with mental diseases [16]. Long-term alcohol abuse can cause multiple medical comorbidities including liver damage because of the inflammatory and oxidative stress induced by alcohol [17].

*IL6* is located on chromosome 7p21 and rs1800795 is a promoter variant affecting IL-6 transcription levels. GG carriers have higher IL-6 serum levels in comparison to CC carriers [18]. Interestingly, this seems to be a gender-dependent genetic predisposition given that only male homozygotes for the G allele have higher IL-6 levels compared to carriers of the C allele [19]. Additionally, in healthy controls, the C allele was associated with significantly lower levels of plasma IL-6 [20]. To our knowledge, this is the first time that an association between *IL6* rs1800795 and alcohol addiction has been revealed. *IL6* rs1800795 was proven to be a risk factor for various diseases, including arteriovenous malformations of the brain [21]. 

Moreover, a whole-brain analysis revealed the neuroprotective role of rs1800795, given that GG carriers had significantly larger hippocampus gray matter volumes than heterozygous carriers. The hippocampus has a critical role in normal brain function, including learning, memory consolidation, and stress and the response to cytokines [18]. Furthermore, the hippocampus is related to various mental disorders, like depression, which does not cause permanent brain damage, but temporary neurotransmitter imbalances [22]. Interestingly, both human and animal studies have shown that heavy alcohol consumption is associated with episodes of alcohol-induced memory loss, blackouts, and functioning impairment, which are possibly linked to hippocampal brain volume loss. Also, a longitudinal magnetic resonance imaging study indicated that heavy drinkers had high rates of grey matter volume decline, which was further related to reduced memory and blackouts [23]. Alcohol consumption can also affect frontal regions and prefrontal white-matter pathways, which are linked to executive functions [24]. Alcohol consumption is associated with a reduced capacity to process new information, develop new skills, formulate, and execute plans and goals [25]. Moreover, IL6 has been associated with cognitive impairment proportionally, i.e., a high IL6 concentration is linked with a high rate of executive and memory function deficit in elderly participants. Furthermore, CC carriers had higher IL-6 concentrations and demonstrated a worse performance in the Stroop cognitive performance test in comparison with the GG carriers. The Stroop test is related to brain function, and measures selective attention capacity and skills, evaluating a person’s overall executive processing abilities and examining the changes in neural activity [26]. 

Taken all together, the association revealed from our study regarding *IL6* rs1800795 and alcohol addiction is of great interest. Potentially, *IL6* rs1800795 influences IL6 expression, which can be further associated with cognitive impairment. Our finding comes as a possible explanation regarding the impairment of several cognitive function domains through brain morphology and connectivity in patients with alcohol addiction. At the same time, it can open the way to the diagnosis and prognosis of patients who are at risk of cognitive function deficits and empower therapeutic interventions to strengthen patients’ ability to abstain from alcohol. 

Our findings also indicated an association between *IL1B* rs1071676 and AUDIT scores. rs1071676 is located in the 3′-UTR region of *IL1B* but its exact function is still unknown. An in silico analysis showed that rs1071676 is located on the target site for has-miR-622 and thus potentially affects miRNA activity [27]. miR-622 has been associated with different types of cancer and cell proliferation, apoptosis, migration, and invasion [28]. 

Higher AUDIT scores are associated with alcohol-clustering conditions and more pronounced alcohol addiction. An AUDIT score equal to or higher than 20 has been associated with depression and anxiety, and the misuse of tobacco or other substances [29]. In our study, the median of patients homozygous for the G allele was five times higher than homozygous controls. This is the first time that an association between rs1071676 and AUDIT scores has been observed. However, a previous cross-sectional assessment of patients with depression aimed to elucidate the role of inflammation in light of previous alcohol use. Patients scoring above the AUDIT cutoff had higher IL-1β serum levels, but with no statistical significance [30]. 

Also, studies show that the heavier the drinking status, the heavier the smoking status [31]. Within our study, we also found an association between *IL6R* rs2228145 and compulsions in alcohol-addicted patients and aggressive symptomatology in healthy individuals. rs2228145 has been associated with decreased IL-6-induced C-reactive protein (CRP) levels [32] and depression severity [33]. Furthermore, high blood CRP and IL6 levels have been associated with greater depression symptom severity, obsessive compulsive disorder (OCD) anxiety and psychological distress [34]. Depressive symptoms were positively correlated with higher overall OCD severity, and higher obsession and compulsion levels. The higher the levels of depressive symptomatology, the higher the levels of counting compulsions, but there was no statistically significant association with specific categories of obsessions or compulsions [35]. Heavy, regular, and binge drinking may be associated with depression due to the brain chemical and homeostatic imbalance caused by alcohol. Alcohol consumption is considered a psychological stimulus that triggers a stress response [36]. Subsequently, this may result in immune system activation and the deregulation of pro-inflammatory cytokines, with CRP thought to be a good representative of this inflammatory response [34]. IL-6 was reported to be elevated in alcohol-addicted patients with alcohol-induced liver and/or pancreatic diseases, and the association between immune mediators and psychiatric comorbidity was noticed in those patients [17,37]. Combining our finding with the available scientific results, it is likely that rs2228145 is the mediator responsible for the compulsory symptoms in alcohol-addicted patients, possibly through a buffering system of IL6 and CRP levels. Genotyping patients for *IL6R* rs2228145 while measuring their IL6 and CRP levels could be useful tools as non-specific diagnostic markers of alcohol- addicted patients with comorbid psychiatric disorders.

Regarding the association, in controls, between *IL6R* rs2228145 and anger, hostility, and aggression, there are no similar findings. 

We also indicated an association between *SOD2* rs4880 and obsessive–compulsive symptoms in alcohol-addicted patients and controls. Although rs4880 is the most studied genetic variant of SOD2, this is the first time that an association between obsessive–compulsive symptoms and alcohol addiction has been revealed.

ROS accumulation and oxidative damage can be affected by SOD2 genetic variability. rs4880 results in an amino acid change, which eventually causes changes in the secondary structure and function of the enzyme. rs4880 can lead to a redox status imbalance by altering enzyme localization and mitochondrial transportation. Individuals with the mutation have increased ROS production, which leads to mitochondrial dysfunction and increased oxidative stress vulnerability [38]. In the presence of rs4880, the translocation of SOD2 into the mitochondria is enhanced and the concentration of the active form of MnSOD is altered [39]. Homozygotes of the C allele have higher enzyme activity, whereas TT carriers have lower SOD2 enzyme activity, and a risk of higher ROS levels, probably due to the low efficiency of enzyme targeting from the cytoplasm to the mitochondria [40]. 

A case–control study using occupational stress as a variable to investigate the role of *SOD2* rs4880 in Han Chinese miners with dyslipidemia found that the condition was associated with occupational stress and genetic factors [41]. 

Moreover, the rs4880 T allele is associated with an increased risk of developing schizophrenia [42] and depression. Interestingly, sex was an influencing factor, given that the depression risk was only in males [40]. However, a large multi-site study investigating the impact of rs4880 on schizophrenia cognitive deficits, while measuring SOD2 activity, indicated that AA homozygotes had poorer attention performance. This allele was also associated with the scores of the Repeatable Battery for the Assessment of Neuropsychological Status scale, which measures cognitive functioning, while indexing, among others, immediate and delayed memory, visuospatial/constructional abilities, and language [43]. 

Additionally, rs4880 is associated with structural differences in the brain regions relevant to BD. More specifically, the caudal anterior cingulate cortex surface area and volume, as well as the prefrontal cortex volume, were lower in GG carriers. Also, GG BD patients had significant interaction effects in a temporal lobe brain region associated with smaller brain structures [42]. Chronic alcohol consumption has been associated with a reduction in gray and white matter volume, which leads to whole-brain volume decline. The shrinking of the brain volume subsequently causes cognitive function deficits in executive and occupational functioning, attention, working, and long-term memory, as well as the poor performance of daily living activities, social skills, and problem-solving in alcohol-addicted patients [44]. The brain is extremely vulnerable to oxidative stress, caused by the reduced concentration of antioxidant enzymes and increased oxygen metabolism [45]. Oxidative stress can damage brain white matter and lead to cognitive function impairment [46].

Alcohol consumption can potentially intensify ROS production in the brain, and induce alcohol-related organ damage by augmenting oxidative damage and neuronal cell death. Through the action of antioxidant enzymes, like SOD2, this imbalance can be shifted, and the production of active radicals can be reduced and further limit the damaging effects on cells. Notably, the low-activity Ala allele was a risk factor only in light and not in heavy drinkers, indicating a protective role of this polymorphism [45]. The involvement of *SOD2* rs4880 may be relevant to the brain changes related to alcohol consumption. 

We found no association between *SOD2* rs4880 and alcohol addiction. However, we did observe an association between this genetic variant and obsessive–compulsive symptoms in alcohol-addicted patients. 

OCD has been linked to increased oxidative stress [47]. Alcohol consumption may lead to an increase of ROS and may thus be connected with obsessive–compulsive symptoms [12]. Patients with anxiety, including OCD, are characterized by an oxidative stress imbalance, due to the dysregulation of free radicals, and DNA damage. Specifically, their antioxidant defense is reduced, and protein, lipid, and DNA damage is high, leading to the dysregulation of cell functions [48]. However, the findings regarding the role of SOD levels as an antioxidant marker in OCD are inconsistent [49,50,51]. SOD levels were statistically significant and higher in OCD patients in comparison with healthy individuals, all males, whose symptoms were assessed using the YBOCS scale. Given that SOD has a protective role against oxidative stress, the authors concluded that oxidative stress may be involved in the pathophysiology of OCD [49]. In contrast, a study investigating the serum antioxidant levels and DNA damage degree found no significant difference in the SOD levels between OCD patients and controls and no associations between the C-YBOCS and depression scores. Overall, OCD patients had increased antioxidant levels and oxidative DNA damage, supporting the involvement of oxidative stress in OCD [50]. In another study, SOD activity levels were higher in OCD patients with concurrent depression in comparison to controls [52]. 

As far as the involvement of *SOD2* rs4880 is concerned, a cross-sectional study demonstrated its potential involvement in OCD by increasing oxidative stress. Notably, rs4880 CT’s frequency was lower and CC was higher in OCD patients than in controls [47]. 

In our case, *SOD2* rs4880 was associated with the YBOCS compulsions and obsessions subscales in both patients and healthy controls, which might be the reason for the inconsistent results in the available literature regarding SOD2 activity levels. 

Our study also revealed an association between *PON1* rs705381 and social phobia. In the controls, *PON1* rs705381 was associated with compulsions, while rs705379 was associated with social phobia, aggression, anxiety, and depression and rs854560 with social phobia, obsessive symptoms, and anxiety. 

PON1 is a calcium-dependent glycoprotein with anti-inflammatory and antioxidant abilities. It reduces oxidized lipids in both low-density lipoprotein and high-density lipoprotein and inhibits their peroxidation [53]. The PON1 enzyme has also been shown to play an important role in aging [54]. PON1 activity can be altered due to various factors, including dietary and lifestyle habits, such as alcohol consumption [55]. Alcohol can inhibit serum PON1 activity [56], but also increases its activity levels, especially in men, possibly through the increase of high-density lipoprotein-cholesterol and apolipoprotein concentrations or the reduction of oxidative stress [57]. 

*PON1* rs705381 is associated with increased serum PON1 levels. In fact, rs705381 is responsible for almost 3% of the variation in PON1 expression levels [53]. rs705381, combined with another variant of the acetylcholinesterase-paraoxonase 1 locus, significantly contributes to the reduced PON1 expression and trait-anxiety measures of healthy subjects [58]. Our study is the first to have investigated and revealed an association between *PON1* rs705381 and social phobia in patients with alcohol addiction and compulsion in healthy individuals. rs705379 contributes almost 23% of the PON1 expression variation, leading to increased serum and plasma PON1 levels [53]. rs705379 was also shown to influence PON1 enzyme activity [59].

In a case–control study on children with attention deficit/hyperactivity disorder (ADHD), GG carriers had low urinary dimethyl phosphate levels [60]. It was reported that one third of hospitalized patients with alcohol addiction have hypophosphatemia, caused by the impairment of phosphate absorption in the intestine due to alcohol consumption [61]. ADHD symptoms include difficulties in concentrating and focusing on organizing and other tasks, as well as hyperactivity and impulsiveness, which can lead to poor social interaction and psychological distress, including anxiety and depression. Although there are no studies investigating the potential association of rs705379 with psychological traits, we found an association between rs705379 and social phobia, aggression, anxiety, and depression in healthy controls. 

rs854560 is a missense variation associated with reduced PON1 concentration, mRNA, and activity levels. Leu carriers have a higher PON1 activity and mRNA and protein levels than the Met homozygotes. Heterozygotes have intermediate levels of mRNA, protein, and gene activity [62]. rs854560′s effect is probably due to the linkage disequilibrium of the SNP with rs705379 [63]. rs854560 has been extensively studied in patients with dyslipidemia, with inconclusive results [64]. Reviewing the available literature, our study is the only one that supports the association between rs854560 and social phobia, obsessive–compulsive, and anxiety symptoms in controls. 

Finally, it should be noted that we found no association between *GPX1* rs1050450, *PON1* rs662, *IL1B* rs1143623, rs16944, and miR146a rs2910164 and alcohol addiction, as well as none with the psychological and behavioral traits examined. 

One of the limitations of our study is its relatively small sample size. Thus, genetic variants with low penetrance which can potentially increase the risk of alcohol addiction might have been missed, despite the ethnically very homogenous genetic background. The alcohol-addicted patients were also older, more frequently smokers and single, less educated, and from rural environment, compared to healthy individuals. However, when we adjusted the genotype distribution for these clinical differences, we found no difference in the statistically significant findings. The other limitation is that the female population was not included in the study. Although the evidence from genetically informed studies suggests that the source and magnitude of genetic influences on alcohol outcomes are likely the same across sexes, there are sex differences in the subjective and neurobiological responses to alcohol intoxication and the genetic factors shared between AUD and endophenotypes such as alcohol sensitivity. Notably, Wigner et al. indicated that the rs4880 TT genotype is associated with an increased risk of depression only in male patients, and suggested that such a result might reflect differences in the regulation of SOD2 enzymatic activity between males and females [40]. By including only the male population, our results are also easier to compare with the other studies, as most clinical and preclinical studies have investigated alcohol exposure in males [65].

## 5. Conclusions

Our pilot study is the first investigating a genetic model of the inflammation- and oxidative stress-related pathways in patients with alcohol addiction and revealing associations with behavioral, psychological, and environmental factors. Our data revealed that *IL6* rs1800795 may play some role in the susceptibility to alcohol addiction, as well as that the genetic variation of both the oxidative stress and inflammation pathways potentially impact the psychosymptomatology of alcohol-addicted patients and healthy individuals. A better understanding of these underlying genetic factors and their interaction with psychosymptomatology may help in the clinical management of alcohol addiction. 

## Figures and Tables

**Table 1:** Cohorts’ characteristics.

Characteristic		Hospitalized Alcohol-Addicted Patients (N = 89)	Healthy Controls (N = 93)	*p* *
Age	years, median (25–75%)	47 (39–54)	36 (26–44.5)	**<0.001**
Education	years, median (25–75%)	12 (11–12)	12 (12–12)	**<0.001**
Partnership	Single, N (%)	38 (42.7)	25 (26.9)	**0.002**
	Partnership, N (%)	51 (57.3)	68 (73.1)	
Environment	Rural, N (%)	46 (51.7)	37 (39.8)	0.137
	Urban, N (%)	43 (48.3)	56 (60.2)	
Smoking	No, N (%)	58 (65.2)	24 (25.8)	**<0.001**
	Yes, N (%)	31 (34.8)	69 (74.2)	

* Fisher’s exact test for categorical variables, Mann–Whitney test for continuous variables. Statistically significant *p*-values are printed in bold.

**Table 2:** Comparison of genotype frequencies of genetic variants of inflammation pathways between hospitalized alcohol-addicted patients and healthy controls.

Gene	SNP	Genotype	Patients (N = 89) N (%)	Controls (N = 93) N (%)	OR (95% CI)	*p*	OR (95% CI)_adj_	P_adj_
*IL1B*	rs1143623	GG	39 (43.8)	49 (53.3)	Reference		Reference	
		GC	45 (50.6)	36 (39.1)	1.57 (0.86–2.88)	0.145	1.15 (0.5–2.64)	0.739
		CC	5 (5.6)	7 (7.6)	0.9 (0.26–3.05)	0.862	0.53 (0.09–3.01)	0.476
		GC + CC	50 (56.2)	43 (46.7)	1.46 (0.81–2.62)	0.205	1.04 (0.47–2.33)	0.915
*IL1B*	rs16944	TT	9 (10.1)	9 (9.8)	Reference		Reference	
		TC	48 (53.9)	38 (41.3)	1.26 (0.46–3.49)	0.653	1.38 (0.34–5.66)	0.656
		CC	32 (36)	45 (48.9)	0.71 (0.25–1.99)	0.516	1.05 (0.25–4.4)	0.942
		TC + CC	80 (89.9)	83 (90.2)	0.96 (0.36–2.55)	0.941	1.22 (0.31–4.74)	0.777
*IL1B*	rs1071676	GG	54 (60.7)	45 (48.9)	Reference		Reference	
		GC	31 (34.8)	37 (40.2)	0.7 (0.38–1.3)	0.256	0.57 (0.25–1.34)	0.200
		CC	4 (4.5)	10 (10.9)	0.33 (0.1–1.13)	0.079	0.34 (0.07–1.66)	0.184
		GC + CC	35 (39.3)	47 (51.1)	0.62 (0.34–1.12)	0.113	0.53 (0.23–1.18)	0.118
*MIRN146A*	rs2910164	GG	51 (57.3)	57 (61.3)	Reference		Reference	
		GC	31 (34.8)	31 (33.3)	1.12 (0.6–2.09)	0.727	0.92 (0.39–2.16)	0.855
		CC	7 (7.9)	5 (5.4)	1.56 (0.47–5.24)	0.468	1.35 (0.23–8)	0.742
		GC + CC	38 (42.7)	36 (38.7)	1.18 (0.65–2.13)	0.584	0.97 (0.43–2.19)	0.947
*IL6*	rs1800795	GG	37 (41.6)	31 (33.7)	Reference		Reference	
		GC	43 (48.3)	41 (44.6)	0.88 (0.46–1.67)	0.693	0.57 (0.23–1.38)	0.210
		CC	9 (10.1)	20 (21.7)	0.38 (0.15–0.95)	**0.038**	0.27 (0.07–0.96)	**0.043**
		GC + CC	52 (58.4)	61 (66.3)	0.71 (0.39–1.31)	0.275	0.47 (0.2–1.1)	0.081
*IL6R*	rs2228145	AA	38 (42.7)	35 (38.5)	Reference		Reference	
		AC	36 (40.4)	40 (44)	0.83 (0.44–1.58)	0.568	0.43 (0.17–1.1)	0.078
		CC	15 (16.9)	16 (17.5)	0.86 (0.37–2)	0.732	0.67 (0.2–2.3)	0.527
		AC + CC	51 (57.3)	56 (61.6)	0.84 (0.46–1.52)	0.563	0.49 (0.21–1.16)	0.105

P_adj:_ adjusted for age, education, smoking, environment, and partnership. Statistically significant *p*-values are printed in bold.

**Table 3:** Associations between studied genes and polymorphisms and the scores of the selected psychosymptomatology scales in hospitalized patients with alcohol addiction.

Alcohol Addiction Comorbid Symptoms (Scale)	*SOD2*	*GPX1*	*PON1*				*IL1B*			*MIRN146A*	*IL6*	*IL6R*
	rs4880	rs1050450	rs705381	rs705379	rs854560	rs662	rs1143623	rs16944	rs1071676	rs2910164	rs1800795	rs2228145
Alcohol addiction severity (AUDIT)	*p* = 0.591	*p* = 0.852	*p* = 0.315	*p* = 0.089	*p* = 0.779	*p* = 0.538	*p* = 0.865	*p* = 0.223	***p* = 0.045 ^#^**	*p* = 0.705	*p* = 0.828	*p* = 0.749
Obsessive-compulsive drinking symptoms (OCDS)	*p* = 0.950	*p* = 0.709	*p* = 0.315	*p* = 0.502	*p* = 0.961	*p* = 0.227	*p* = 0.313	*p* = 0.688	*p* = 0.804	*p* = 0.891	*p* = 0.543	*p* = 0.542
Obsessions (YBOCS)	***p* = 0.016**	*p* = 0.899	*p* = 0.965	*p* = 0.570	*p* = 0.520	*p* = 0.951	*p* = 0.758	*p* = 0.834	*p* = 0.416	*p* = 0.236	*p* = 0.259	*p* = 0.467
Compulsions (YBOCS)	***p* = 0.046**	*p* = 0.742	*p* = 0.457	*p* = 0.437	*p* = 0.592	*p* = 0.376	*p* = 0.645	*p* = 0.282	*p* = 0.627	*p* = 0.580	*p* = 0.487	***p* = 0.033 ^#^**
Social phobia (BSPS)	*p* = 0.591	*p* = 0.181	***p* = 0.041**	*p* = 0.555	*p* = 0.486	*p* = 0.935	*p* = 0.778	*p* = 0.624	*p* = 0.923	*p* = 0.826	*p* = 0.815	*p* = 0.104
Depression (Zung)	*p* = 0.915	*p* = 0.150	*p* = 0.250	*p* = 0.938	*p* = 0.415	*p* = 0.921	*p* = 0.451	*p* = 0.513	*p* = 0.626	*p* = 0.222	*p* = 0.382	*p* = 0.517
Anxiety (Zung)	*p* = 0.900	*p* = 0.753	*p* = 0.743	*p* = 0.866	*p* = 0.115	*p* = 0.438	*p* = 0.763	*p* = 0.723	*p* = 0.050	*p* = 0.842	*p* = 0.705	*p* = 0.558
Aggression (BDHI)	*p* = 0.685	*p* = 0.405	*p* = 0.188	*p* = 0.895	*p* = 0.165	*p* = 0.353	*p* = 0.449	*p* = 0.327	*p* = 0.380	*p* = 0.983	*p* = 0.832	*p* = 0.595

SOD2: superoxide dismutase 2, GPX1: glutathione peroxidase 1, PON1: paraoxonase-11, IL1B: interleukin-1β, IL6: interleukin-6, IL6R: IL-6 receptor, YBOCS: Yale-Brown Obsessive Compulsive Scale, BSPS: Brief Social Phobia Scale, AUDIT: Alcohol Use Disorders Identification Test, OCDS: Obsessive Compulsive Drinking Scale, BDHI: Buss-Durkee Hostility Inventory. The *p* values are shown for the dominant genetic model, except for those marked with ^#^, which are for the additive model. Statistically significant *p*-values are printed in bold.

**Table 4:** Associations between studied genes and polymorphisms and the scores of the selected psychosymptomatology scales in healthy controls.

Alcohol Addiction Comorbid Symptoms (Scale)	*SOD2*	*GPX1*	*PON1*				*IL1B*			*MIRN146A*	*IL6*	*IL6R*
	rs4880	rs1050450	rs705381	rs705379	rs854560	rs662	rs1143623	rs16944	rs1071676	rs2910164	rs1800795	rs2228145
Alcohol addiction severity (AUDIT)	*p* = 0.847	*p* = 0.254	*p* = 0.440	*p* = 0.489	*p* = 0.065	*p* = 0.229	*p* = 0.294	*p* = 0.775	*p* = 0.770	*p* = 0.518	*p* = 0.057	*p* = 0.088
Obsessive-compulsive drinking symptoms (OCDS)	*p* = 0.907	*p* = 0.564	*p* = 0.973	*p* = 0.795	*p* = 0.293	*p* = 0.895	*p* = 0.483	*p* = 0.822	*p* = 0.907	*p* = 0.626	*p* = 0.187	*p* = 0.269
Obsessions (YBOCS)	*p* = 0.338	*p* = 0.110	*p* = 0.445	*p* = 0.055	***p* = 0.038 ^#^**	*p* = 0.414	*p* = 0.503	*p* = 0.082	*p* = 0.342	*p* = 0.478	*p* = 0.190	*p* = 0.439
Compulsions (YBOCS)	*p* = 0.196	*p* = 0.584	***p* = 0.027**	*p* = 0.097	*p* = 0.159	*p* = 0.976	*p* = 0.312	*p* = 0.167	*p* = 0.166	*p* = 0.315	*p* = 0.483	*p* = 0.204
Social phobia (BSPS)	*p* = 0.108	*p* = 0.133	*p* = 0.083	***p* = 0.002**	***p* = 0.014**	*p* = 0.151	*p* = 0.284	*p* = 0.921	*p* = 0.431	*p* = 0.382	*p* = 0.305	*p* = 0.313
Depression (Zung)	*p* = 0.073	*p* = 0.707	*p* = 0.873	***p* = 0.001**	*p* = 0.108	*p* = 0.751	*p* = 0.715	*p* = 0.584	*p* = 0.665	*p* = 0.630	*p* = 0.923	*p* = 0.096
Anxiety (Zung)	*p* = 0.373	*p* = 0.728	*p* = 0.271	***p* = 0.001**	***p* = 0.003**	*p* = 0.614	*p* = 0.883	*p* = 0.360	*p* = 0.662	*p* = 0.402	*p* = 0.260	*p* = 0.465
Aggression (BDHI)	*p* = 0.662	*p* = 0.735	*p* = 0.357	***p* = 0.047**	*p* = 0.331	*p* = 0.743	*p* = 0.959	*p* = 0.948	*p* = 0.664	*p* = 0.339	*p* = 0.990	***p* = 0.004**

SOD2: superoxide dismutase 2, GPX1: glutathione peroxidase 1, PON1: paraoxonase-11, IL1B interleukin-1β, IL6: interleukin-6, IL6R: IL-6 receptor, YBOCS: Yale-Brown Obsessive Compulsive Scale, BSPS: Brief Social Phobia Scale, AUDIT: Alcohol Use Disorders Identification Test, OCDS: Obsessive Compulsive Drinking Scale, BDHI: Buss-Durkee Hostility Inventory. The *p* values are shown for the dominant genetic model, except for those marked with ^#^, which are for the additive model. Statistically significant *p*-values are printed in bold.

## Data Availability

All the supporting data are reported in the Supplementary Files. All relevant raw data presented in this study are available on request from the corresponding authors. The raw data are not publicly available due to ethical restrictions.

## Supplementary Materials

The following supporting information can be downloaded at https://www.mdpi.com/article/10.3390/antiox13010020/s1, Table S1: questionnaire traits and scores, Table S2: the investigated genetic variants, their minor allele frequencies, and Hardy–Weinberg Equilibrium in controls, Table S3: comparison of genotype frequencies of genetic variants of oxidative stress pathways between hospitalized alcohol-addicted patients and healthy controls, Table S4: associations between SOD2 rs4880 and the selected psychosymptomatology scale scores for hospitalized alcohol-dependent patients and healthy controls, Table S5: associations between GPX1 rs1050450 and the selected psychosymptomatology scale scores for hospitalized alcohol-dependent patients and healthy controls, Table S6: associations between PON1 rs705381 and the selected psychosymptomatology scale scores for hospitalized alcohol-dependent patients and healthy controls, Table S7: associations between PON1 rs705379 and the selected psychosymptomatology scale scores for hospitalized alcohol-dependent patients and healthy controls, Table S8: associations between PON1 rs854560 and the selected psychosymptomatology scale scores for hospitalized alcohol-dependent patients and healthy controls, Table S9: associations between PON1 rs662 and the selected psychosymptomatology scale scores for hospitalized alcohol-dependent patients and healthy controls, Table S10: associations between IL1B rs1143623 and the selected psychosymptomatology scale scores for hospitalized alcohol-dependent patients and healthy controls, Table S11: associations between IL1B rs16944 and the selected psychosymptomatology scale scores for hospitalized alcohol-dependent patients and healthy controls, Table S12: associations between IL1B rs1071676 and the selected psychosymptomatology scale scores for hospitalized alcohol-dependent patients and healthy controls, Table S13: associations between MIRN146A rs2910164 and the selected psychosymptomatology scale scores for hospitalized alcohol-dependent patients and healthy controls, Table S14: associations between IL6 rs1800795 and the selected psychosymptomatology scale scores for hospitalized alcohol-dependent patients and healthy controls, Table S15: associations between IL6R rs2228145 and the selected psychosymptomatology scale scores for hospitalized alcohol-dependent patients and healthy controls, Table S16: thermal cycling conditions used for GPX1 rs1050450, IL1β rs1143623 and rs16944, IL6 rs1800795, IL6R rs2228145, miR146a rs2910164, PON1 rs854560 and rs662, and SOD2 rs4880 genotyping, Table S17: thermal cycling conditions used for PON1 rs705379 and rs705381 genotyping, Table S18: thermal cycling conditions used for IL1β rs1071676 genotyping.
