# Supplementary material for: The Disease Model of Addiction: The Impact of Genetic Variability in the Oxidative Stress and Inflammation Pathways on Alcohol Dependance and Comorbid Psychosymptomatology
Source: Antioxidants (Basel). 2023 Dec 21;13(1):20. doi: 10.3390/antiox13010020 (PMC10812813; doi:10.3390/antiox13010020)
Supplement: Supplementary file 1 [file antioxidants-13-00020-s001.zip › antioxidants-2740513-supplementary.pdf]

The Disease Model of Addiction: The Impact of Genetic Variability in the Oxidative Stress and Inflammation Pathways on Alcohol Dependance and Comorbid Psychosymptomatology

Evangelia Eirini Tsermpini [1], Katja Goričar [1], Blanka Kores Plesničar [2,3], Anja Plemenitaš Ilješ [4,*] and Vita Dolžan [1,*]

[1] Pharmacogenetics Laboratory, Institute of Biochemistry and Molecular Genetics, Faculty of Medicine, University of Ljubljana, 1000 Ljubljana, Slovenia; evangelia.tsermpini@dal.ca (E.E.T.); katja.goricar@mf.uni-lj.si (K.G.)
[2] University Psychiatric Clinic, 1000 Ljubljana, Slovenia; blanka.kores@psih-klinika.si
[3] Faculty of Medicine, University of Ljubljana, 1000 Ljubljana, Slovenia
[4] Department of Psychiatry, University Clinical Centre Maribor, 2000 Maribor, Slovenia
* Correspondence: anja.plemenitas@ukc-mb.si (A.P.I.); vita.dolzan@mf.uni-lj.si (V.D.)

Supplementary material

Supplementary Table S1. Questionnaire traits and scores.

**Trait**	**Questionnaire**	**Hospitalized alcohol-dependent patients (N=89)** points, median (25-75%)	**Healthy controls (N=93)** points, median (25-75%)	**P***
Obsession	YBOCS obsession	2 (1-7)	1 (1-1)	<0.001
Compulsion	YBOCS compulsion	1 (1-3)	1 (1-1)	<0.001
Social phobia	BSPS	10 (4-18.5)	9 (5.5-14)	0.693
Alcohol-dependence severity	AUDIT	23 (19-28.5)	5 (4-7)	<0.001
Obsessive-compulsive drinking	OCDS	18 (9-26.5)	3 (2-4)	<0.001
Depression	Zung depression	34 (27-45)	22 (20-24)	<0.001
Anxiety	Zung anxiety	34 (29-39)	22 (20-24)	<0.001
Anger, hostility and aggression	BDHI	30 (22-40)	17 (10.5-23)	<0.001

* Mann-Whitney test

Supplementary Table S2. The investigated genetic variants, their minor allele frequencies, and Hardy-Weinberg Equilibrium in controls.

Gene	SNP	Genotype	MAF (controls) (%)	P_{HWE} (controls)
SOD2	rs4880[a]	CC	39.0	0.415
	p.Ala16Val	CT		
		TT		
GPX1	rs1050450[b]	CC	31.9	0.714
	p.Pro198Leu	CT		
		TT		
PON1	rs705381[c]	CC	22.3	0.389
	c.-162C>T	CT		
		TT		
PON1	rs705379[c]	GG	52.2	0.986
	c.-108G>A	GA		
		AA		
PON1	rs854560[c]	AA	35.3	0.813
	p.Leu55Met	AT		
		TT		
PON1	rs662	AA	29.6	0.289
	p.Gln192Arg	AG		
		GG		
IL1B	rs1143623[c]	GG	30.9	0.083
	c.-1560G>C	GC		
		CC		
IL1B	rs16944[c]	TT	69.6	0.814
	c.-598T>C	TC		
		CC		
IL1B	rs1071676[c]	GG	31.0	0.568
	c.*505G>C	GC		
		CC		
MIRN146A	rs2910164	GG	22.0	0.772
	n.60G>C	GC		
		CC		
IL6	rs1800795[c]	GG	44.0	0.358
	c.-174G>C	GC		
		CC		
IL6R	rs2228145[a]	AA	39.6	0.441
	p.Asp358Ala	AC		
		CC		

[a]data missing for 2 controls, [b]data missing for 2 controls and 2 alcoholics, [c]data missing for 1 control

Supplementary Table S3. Comparison of genotype frequencies of genetic variants of oxidative stress pathways between hospitalized alcohol-addicted patients and healthy controls.

Gene	SNP	Genotype	Patients (N=89) N (%)	Controls (N=93) N (%)	OR (95% CI)	P	OR (95% CI)$_{adj}$	P$_{adj}$
SOD2	rs4880	CC	21 (23.6)	32 (35.2)	Reference		Reference	
		CT	52 (58.4)	47 (51.6)	1.69 (0.86-3.32)	0.131	1.34 (0.51-3.46)	0.552
		TT	16 (18)	12 (13.2)	2.03 (0.8-5.14)	0.135	1.54 (0.47-5.04)	0.471
		CT+TT	68 (76.4)	59 (64.8)	1.76 (0.92-3.37)	0.090	1.39 (0.57-3.43)	0.472
GPX1	rs1050450	CC	44 (50.6)	43 (47.3)	Reference		Reference	
		CT	38 (43.7)	38 (41.8)	0.98 (0.53-1.81)	0.942	1.66 (0.7-3.92)	0.249
		TT	5 (5.7)	10 (11)	0.49 (0.15-1.55)	0.223	0.59 (0.12-2.79)	0.502
		CT+TT	43 (49.4)	48 (52.8)	0.88 (0.49-1.58)	0.658	1.4 (0.62-3.16)	0.420
PON1	rs705381	CC	51 (57.3)	57 (62)	Reference		Reference	
		CT	30 (33.7)	29 (31.5)	1.16 (0.61-2.18)	0.654	0.87 (0.36-2.12)	0.758
		TT	8 (9)	6 (6.5)	1.49 (0.48-4.59)	0.487	0.66 (0.14-3.09)	0.599
		CT+TT	38 (42.7)	35 (38)	1.21 (0.67-2.2)	0.524	0.82 (0.36-1.9)	0.649
PON1	rs705379	GG	25 (28.1)	21 (22.8)	Reference		Reference	
		GA	47 (52.8)	46 (50)	0.86 (0.42-1.74)	0.672	1.52 (0.53-4.33)	0.431
		AA	17 (19.1)	25 (27.2)	0.57 (0.25-1.33)	0.195	0.71 (0.22-2.32)	0.570
		GA+AA	64 (71.9)	71 (77.2)	0.76 (0.39-1.48)	0.417	1.18 (0.44-3.14)	0.746
PON1	rs854560	AA	41 (46.1)	39 (42.4)	Reference		Reference	
		AT	38 (42.7)	41 (44.6)	0.88 (0.47-1.64)	0.691	1.08 (0.45-2.58)	0.862
		TT	10 (11.2)	12 (13)	0.79 (0.31-2.04)	0.631	1.97 (0.53-7.23)	0.309
		AT+TT	48 (53.9)	53 (57.6)	0.86 (0.48-1.55)	0.619	1.23 (0.54-2.8)	0.623
PON1	rs662	AA	45 (50.6)	44 (47.3)	Reference		Reference	
		AG	34 (38.2)	43 (46.2)	0.77 (0.42-1.43)	0.410	0.47 (0.2-1.14)	0.094
		GG	10 (11.2)	6 (6.5)	1.63 (0.55-4.87)	0.382	2.03 (0.44-9.29)	0.362
		AG+GG	44 (49.4)	49 (52.7)	0.88 (0.49-1.57)	0.661	0.62 (0.28-1.4)	0.252

Adj: adjusted for age, education, smoking, environment, and partnership

Supplementary Table S4. Associations between *SOD2* rs4880 and the selected psychosymptomatology scale scores for hospitalized alcohol-dependent patients and healthy controls.

Scale	*SOD2* rs4880 genotype	Patients (N=89)		Controls (N=93)	
		Median (25-75%)	P*	Median (25-75%)	P*
YBOCS obsession	CC	1 (1-2)	0.054	1 (1-1)	0.607
	CT	2 (1-9)		1 (1-2)	
	TT	3 (1-6.8)		1 (1-1.8)	
	CT+TT	2 (1-8.8)	**0.016**	1 (1-2)	0.338
YBOCS compulsion	CC	1 (1-2)	0.130	1 (1-1)	0.236
	CT	1 (1-6)		1 (1-1)	
	TT	2 (1-5.3)		1 (1-1)	
	CT+TT	1 (1-6)	**0.046**	1 (1-1)	0.196
BSPS	CC	9 (3-16.5)	0.790	7 (4.3-10.8)	0.229
	CT	9.5 (4-19)		10 (7-15)	
	TT	10 (4-17)		8.5 (4.5-17.8)	
	CT+TT	10 (4-19)	0.591	10 (6-15)	0.108
AUDIT	CC	27 (19-28)	0.866	5 (3-7)	0.925
	CT	23 (19-30)		5 (4-7)	
	TT	23.5 (21-26.8)		5 (4-7.5)	
	CT+TT	23 (19.3-29)	0.591	5 (4-7)	0.847
OCDS	CC	16 (12-29)	0.671	3 (2-4)	0.727
	CT	17 (8-26)		3 (2-4)	
	TT	18.5 (14.3-27.8)		3.5 (2.3-4.8)	
	CT+TT	18 (8.3-26.8)	0.950	3 (2-4)	0.907
Zung depression	CC	32 (26-48)	0.992	21 (20-22.8)	0.180
	CT	35 (28-43.8)		22 (21-25)	
	TT	32.5 (25.5-47.3)		22 (20.3-23.8)	
	CT+TT	34 (27.3-43.8)	0.915	22 (21-25)	0.073
Zung anxiety	CC	33 (28.5-38)	0.835	21.5 (20.3-23)	0.668
	CT	34 (29-39.8)		22 (20-24)	
	TT	33.5 (31-45.5)		21.5 (20-26)	
	CT+TT	34 (29-39.8)	0.900	22 (20-25)	0.373
BDHI	CC	34 (22.5-40.5)	0.749	14.5 (10.3-21)	0.101
	CT	30 (22-41)		18 (12-26)	
	TT	29.5 (22.3-35.8)		10.5 (7-20.8)	
	CT+TT	30 (22-39.5)	0.685	17 (10-25)	0.662

*Kruskal-Wall test for additive and Mann-Whitney test for dominant model

Supplementary Table S5. Associations between *GPX1* rs1050450 and the selected psychosymptomatology scale scores for hospitalized alcohol-dependent patients and healthy controls.

**Scale**	***GPX1* rs1050450 genotype**	**Patients (N=89)**		**Controls (N=93)**	
		Median (25-75%)	P*	Median (25-75%)	P*
YBOCS obsession	CC	2 (1-6)	0.507	1 (1-2)	0.103
	CT	1.5 (1-9)		1 (1-1)	
	TT	1 (1-2)		1 (1-1)	
	CT+TT	1 (1-7)	0.899	1 (1-1)	0.110
YBOCS compulsion	CC	1 (1-3)	0.500	1 (1-1)	0.184
	CT	1 (1-2.8)		1 (1-1.3)	
	TT	1 (1-1.5)		1 (1-1)	
	CT+TT	1 (1-2)	0.742	1 (1-1)	0.584
BSPS	CC	11 (5-19)	0.237	7 (4-14)	0.161
	CT	8 (3-16.3)		10.5 (6.8-17.3)	
	TT	4 (3-9.5)		7.5 (5.8-10.8)	
	CT+TT	7 (3-14)	0.181	10 (6.3-14.8)	0.133
AUDIT	CC	23 (20-27.8)	0.447	5 (4-7)	0.352
	CT	24.5 (19-29)		4 (3-7)	
	TT	17 (16.5-26.5)		5 (4-7)	
	CT+TT	23 (19-29)	0.852	4 (3-6.8)	0.254
OCDS	CC	18 (12-26)	0.858	3 (2-4)	0.216
	CT	16.5 (6-31)		3 (2-4)	
	TT	16 (15-25.5)		3.5 (3-5.3)	
	CT+TT	16 (7-29)	0.709	3 (2-4)	0.564
Zung depression	CC	31.5 (25-41.8)	0.317	22 (20-25)	0.862
	CT	36.5 (28.8-47.3)		22 (20.8-24)	
	TT	32 (29-41.5)		22.5 (21-24.5)	
	CT+TT	35 (29-47)	0.150	22 (21-24)	0.707
Zung anxiety	CC	34 (29.3-39.8)	0.592	22 (20-24)	0.607
	CT	34 (28-39.3)		21 (20.8-24)	
	TT	31 (28.5-34)		22.5 (21-24.3)	
	CT+TT	33 (28-38)	0.753	21.5 (21-24)	0.728
BDHI	CC	35 (20-41)	0.707	15 (10-21)	0.943
	CT	29 (22-38.8)		18 (10.8-23.5)	
	TT	33 (23-37)		16.5 (10-25.8)	
	CT+TT	29 (22-38)	0.405	18 (10.3-24.5)	0.735

*Kruskal-Wall test for additive and Mann-Whitney test for dominant model

Supplementary Table S6. Associations between *PON1* rs705381 and the selected psychosymptomatology scale scores for hospitalized alcohol-dependent patients and healthy controls.

**Scale**	***PON1* rs705381 genotype**	**Patients (N=89)**		**Controls (N=93)**	
		Median (25-75%)	P*	Median (25-75%)	P*
YBOCS obsession	CC	2 (1-7)	0.919	1 (1-2)	0.704
	CT	1.5 (1-7)		1 (1-1)	
	TT	2 (1-9.5)		1 (1-1.3)	
	CT+TT	2 (1-7)	0.965	1 (1-1)	0.445
YBOCS compulsion	CC	1 (1-2)	0.359	1 (1-1)	0.081
	CT	2 (1-5.3)		1 (1-1)	
	TT	1 (1-4.8)		1 (1-1)	
	CT+TT	1 (1-5.3)	0.457	1 (1-1)	**0.027**
BSPS	CC	13 (5-19)	0.116	10 (6.5-14.5)	0.192
	CT	6.5 (2-17)		7 (4-14)	
	TT	6.5 (3.5-19.5)		6 (4-11)	
	CT+TT	6.5 (3-17)	**0.041**	7 (4-13)	0.083
AUDIT	CC	23 (19-29)	0.274	5 (4-7)	0.701
	CT	26.5 (20.5-28.5)		4 (3-7.5)	
	TT	21.5 (16.3-27.3)		4.5 (4-5)	
	CT+TT	25.5 (19-28)	0.315	4 (3-7)	0.440
OCDS	CC	19 (10-28)	0.591	3 (2-4)	0.945
	CT	17.5 (6-27.3)		3 (2-5.5)	
	TT	18 (15.5-18.8)		3 (2.8-3.3)	
	CT+TT	18 (7.8-22.3)	0.315	3 (2-5)	0.973
Zung depression	CC	36 (28-45)	0.261	22 (20-24.5)	0.520
	CT	31 (25-36.5)		22 (21-24.5)	
	TT	41 (26.8-47.3)		21 (20.8-22.3)	
	CT+TT	32 (25.8-45)	0.250	22 (21-24)	0.873
Zung anxiety	CC	34 (29-39)	0.796	22 (20.5-24)	0.133
	CT	33 (28-38.5)		21 (20.5-25)	
	TT	34.5 (32.3-36)		20.5 (20-21.3)	
	CT+TT	33.5 (28-38)	0.743	21 (20-24)	0.271
BDHI	CC	32 (23-41)	0.383	18 (10-25.5)	0.051
	CT	28 (20.5-35.8)		17 (12-21)	
	TT	34 (20.5-37.8)		8 (5.8-14.3)	
	CT+TT	29 (20.5-37.3)	0.188	14 (11-21)	0.357

*Kruskal-Wall test for additive and Mann-Whitney test for dominant model

Supplementary Table S7. Associations between *PON1* rs705379 and the selected psychosymptomatology scale scores for hospitalized alcohol-dependent patients and healthy controls.

**Scale**	***PON1* rs705379 genotype**	**Patients (N=89)**		**Controls (N=93)**	
		Median (25-75%)	P*	Median (25-75%)	P*
YBOCS obsession	GG	1 (1-6)	0.825	1 (1-1)	0.078
	GA	2 (1-7)		1 (1-1.3)	
	AA	2 (1-7)		1 (1-3)	
	GA+AA	2 (1-7)	0.570	1 (1-2)	0.055
YBOCS compulsion	GG	1 (1-4.5)	0.671	1 (1-1)	0.159
	GA	1 (1-3)		1 (1-1)	
	AA	2 (1-5.5)		1 (1-1.5)	
	GA+AA	1 (1-3)	0.437	1 (1-1)	0.097
BSPS	GG	9 (3.5-15.5)	0.749	6 (4-8.5)	**0.001**
	GA	10 (3-19)		9.5 (6-13.3)	
	AA	10 (6.5-18.5)		13 (8-22)	
	GA+AA	10 (4-19)	0.555	10 (7-17)	**0.002**
AUDIT	GG	23 (15.5-28)	0.174	5 (4-5)	0.784
	GA	24 (20-29)		5 (3-7)	
	AA	26 (22-29.5)		5 (3.5-6.5)	
	GA+AA	24.5 (21-29)	0.089	5 (3-7)	0.489
OCDS	GG	18 (6.5-24.5)	0.607	3 (2.5-4)	0.944
	GA	18 (9-26)		3 (2-4)	
	AA	26 (11.5-30)		3 (2-4)	
	GA+AA	18 (10.3-27.8)	0.502	3 (2-4)	0.795
Zung depression	GG	32 (27.5-46.5)	0.896	21 (20-22)	**0.005**
	GA	35 (25-45)		22 (21-25)	
	AA	35 (28-43)		22 (20-27)	
	GA+AA	35 (27-44)	0.938	22 (21-26)	**0.001**
Zung anxiety	GG	34 (28.5-39.5)	0.646	21 (20-21.5)	**0.002**
	GA	33 (29-39)		22 (21-24.3)	
	AA	34 (30.5-40)		23 (21-26)	
	GA+AA	33.5 (29.3-39)	0.866	22 (21-25)	**0.001**
BDHI	GG	29 (25-40)	0.421	14 (7.5-16.5)	**0.040**
	GA	30 (22-38)		20 (12.8-25)	
	AA	36 (21-43.5)		14 (8-24.5)	
	GA+AA	31 (22-40)	0.895	19 (11-25)	**0.047**

*Kruskal-Wall test for additive and Mann-Whitney test for dominant model

Supplementary Table S8. Associations between *PON1* rs854560 and the selected psychosymptomatology scale scores for hospitalized alcohol-dependent patients and healthy controls.

**Scale**	***PON1* rs854560 genotype**	**Patients (N=89)**		**Controls (N=93)**	
		Median (25-75%)	P*	Median (25-75%)	P*
YBOCS obsession	AA	2 (1-5)	0.692	1 (1-1)	**0.038**
	AT	2 (1-9)		1 (1-1.5)	
	TT	1 (1-8.5)		1.5 (1-4)	
	AT+TT	2 (1-8.8)	0.520	1 (1-2)	0.095
YBOCS compulsion	AA	1 (1-4)	0.412	1 (1-1)	0.341
	AT	1.5 (1-4)		1 (1-1)	
	TT	1 (1-2.8)		1 (1-1.8)	
	AT+TT	1 (1-3)	0.592	1 (1-1)	0.159
BSPS	AA	9 (4-17)	0.753	7 (4-10)	**0.018**
	AT	10.5 (3.8-19)		10 (6-13.5)	
	TT	11 (5-20.3)		14.5 (7.3-22)	
	AT+TT	10.5 (4-19)	0.486	10 (7-15)	**0.014**
AUDIT	AA	24 (19-28)	0.259	4 (3-5)	0.181
	AT	25.5 (20.8-30)		5 (4-7.5)	
	TT	21 (18.3-24)		5 (3.3-7)	
	AT+TT	23 (19.3-29)	0.779	5 (4-7)	0.065
OCDS	AA	18 (9-24.5)	0.716	3 (2-4)	0.515
	AT	18 (10.8-28.3)		3 (2-4)	
	TT	12 (7.5-26)		3.5 (2-6)	
	AT+TT	17 (8.5-27.8)	0.961	3 (2-4)	0.293
Zung depression	AA	35 (28.5-45)	0.147	22 (20-23)	0.094
	AT	35 (27-48)		22 (20-24.5)	
	TT	29.5 (25-31.8)		23.5 (22-27)	
	AT+TT	32.5 (27-44.3)	0.415	22 (20.5-25.5)	0.108
Zung anxiety	AA	35 (29.5-43)	0.225	21 (20-22)	**0.005**
	AT	33.5 (28-39)		22 (20-25.5)	
	TT	32 (29.8-33.3)		23.5 (21.3-28)	
	AT+TT	32 (29-37.5)	0.115	23 (21-26)	**0.003**
BDHI	AA	33 (26-40.5)	0.091	15 (11-21)	0.176
	AT	32 (21.8-41)		17 (10-23)	
	TT	21 (14-31.3)		21.5 (14.3-33)	
	AT+TT	29 (20.3-39.5)	0.165	18 (10-25.5)	0.331

*Kruskal-Wall test for additive and Mann-Whitney test for dominant model

Supplementary Table S9. Associations between *PON1* rs662 and the selected psychosymptomatology scale scores for hospitalized alcohol-dependent patients and healthy controls.

**Scale**	***PON1* rs662 genotype**	**Patients (N=89)**		**Controls (N=93)**	
		Median (25-75%)	P*	Median (25-75%)	P*
YBOCS obsession	AA	1 (1-9)	0.818	1 (1-2)	0.340
	AG	2 (1-6)		1 (1-1)	
	GG	2.5 (1-8)		1 (1-1)	
	AG+GG	2 (1-6)	0.951	1 (1-1)	0.414
YBOCS compulsion	AA	1 (1-4)	0.619	1 (1-1)	0.520
	AG	1.5 (1-3)		1 (1-1)	
	GG	1.5 (1-8)		1 (1-1)	
	AG+GG	1.5 (1-3)	0.376	1 (1-1)	0.976
BSPS	AA	9 (4-19)	0.986	10 (6.3-17.3)	0.348
	AG	10 (3.8-19)		8 (5-13)	
	GG	12.5 (4.8-16.3)		8 (6.8-11.8)	
	AG+GG	11.5 (4-17.8)	0.935	8 (5-13)	0.151
AUDIT	AA	23 (19.5-29.5)	0.542	5 (4-7)	0.410
	AG	24.5 (19-28.3)		4 (3-6)	
	GG	22 (15.8-25.5)		5 (4.8-5.3)	
	AG+GG	23 (19-28)	0.538	5 (3.5-6)	0.229
OCDS	AA	17 (7-26)	0.449	3 (2-4)	0.413
	AG	17 (12-30.3)		3 (2-4)	
	GG	20.5 (15-26)		2 (2-4)	
	AG+GG	18.5 (12.5-29.3)	0.227	3 (2-4)	0.895
Zung depression	AA	32 (27.5-43.5)	0.911	22 (20-24.8)	0.937
	AG	35 (25-47.3)		22 (20-24)	
	GG	32.5 (28.5-44)		22.5 (20.8-23.3)	
	AG+GG	34.5 (25.5-45.8)	0.921	22 (20.5-24)	0.751
Zung anxiety	AA	32 (29.5-37.5)	0.563	21 (21-24)	0.395
	AG	35 (29-39.3)		22 (20-24)	
	GG	33.5 (26.5-42.5)		20.5 (20-22.5)	
	AG+GG	35 (29-39.8)	0.438	22 (20-23.5)	0.614
BDHI	AA	29 (19.5-39)	0.644	17.5 (10.3-23.5)	0.944
	AG	33 (24.8-41)		15 (10-23)	
	GG	30 (26.5-40.8)		16.5 (12.5-21.8)	
	AG+GG	31 (25.3-40.8)	0.353	15 (10.5-23)	0.743

*Kruskal-Wall test for additive and Mann-Whitney test for dominant model

Supplementary Table S10. Associations between *IL1B* rs1143623 and the selected psychosymptomatology scale scores for hospitalized alcohol-dependent patients and healthy controls.

**Scale**	***IL1B* rs1143623 genotype**	**Patients (N=89)**		**Controls (N=93)**	
		Median (25-75%)	P*	Median (25-75%)	P*
YBOCS obsession	GG	2 (1-6)	0.657	1 (1-2)	0.309
	GC	2 (1-8.5)		1 (1-1.8)	
	CC	1 (1-4.5)		1 (1-1)	
	GC+CC	2 (1-7.3)	0.758	1 (1-1)	0.503
YBOCS compulsion	GG	1 (1-3)	0.778	1 (1-1)	0.386
	GC	1 (1-5.5)		1 (1-1)	
	CC	1 (1-2.5)		1 (1-1)	
	GC+CC	1 (1-5)	0.645	1 (1-1)	0.312
BSPS	GG	14 (3-19)	0.587	9 (7-13.5)	0.564
	GC	9 (4-15)		7 (4-16.5)	
	CC	17 (8-18)		10 (4-13)	
	GC+CC	9 (4-17)	0.778	7 (4-15)	0.284
AUDIT	GG	23 (20-29)	0.366	5 (4-7)	0.447
	GC	23 (19-27.5)		4.5 (3-6.5)	
	CC	28 (23-32)		5 (4-7)	
	GC+CC	23 (19-28)	0.865	5 (3-7)	0.294
OCDS	GG	16 (7-23)	0.561	3 (2-4)	0.694
	GC	18 (11.5-27.5)		3 (2-5)	
	CC	18 (11.5-32)		3 (3-4)	
	GC+CC	18 (11.8-28.3)	0.313	3 (2-5)	0.483
Zung depression	GG	36 (29-46)	0.189	22 (20.5-24.5)	0.890
	GC	31 (26.5-42.5)		22 (20-24.8)	
	CC	41 (33-50.5)		22 (20-23)	
	GC+CC	32 (27-43.5)	0.451	22 (20-24)	0.715
Zung anxiety	GG	34 (29-39)	0.296	22 (20.5-24)	0.864
	GC	33 (30-38)		21.5 (20-23.8)	
	CC	44 (32-49)		22 (20-26)	
	GC+CC	33 (30-39.3)	0.763	22 (20-24)	0.883
BDHI	GG	29 (22-40)	0.430	18 (10-23)	0.897
	GC	34 (20.5-40)		15 (11-23)	
	CC	36 (28-46.5)		17 (8-28)	
	GC+CC	34.5 (21-40.3)	0.449	15 (11-23)	0.959

*Kruskal-Wall test for additive and Mann-Whitney test for dominant model

Supplementary Table S11. Associations between *IL1B* rs16944 and the selected psychosymptomatology scale scores for hospitalized alcohol-dependent patients and healthy controls.

Scale	*IL1B* rs16944 genotype	Patients (N=89)		Controls (N=93)	
		Median (25-75%)	P*	Median (25-75%)	P*
YBOCS obsession	TT	2 (1-8.5)	0.978	1 (1-1)	0.204
	TC	1.5 (1-7)		1 (1-2)	
	CC	2 (1-6)		1 (1-1.5)	
	TC+CC	2 (1-6.8)	0.834	1 (1-2)	0.082
YBOCS compulsion	TT	2 (1-7.5)	0.547	1 (1-1)	0.362
	TC	1 (1-4.3)		1 (1-1)	
	CC	1 (1-2.8)		1 (1-1)	
	TC+CC	1 (1-2.8)	0.282	1 (1-1)	0.167
BSPS	TT	9 (4-17)	0.726	10 (4.5-13.5)	0.606
	TC	9 (4-16.8)		7 (4-15)	
	CC	14 (4-19.8)		9 (6.5-14.5)	
	TC+CC	10 (4-19)	0.624	9 (6-15)	0.921
AUDIT	TT	27 (22-30.5)	0.293	5 (3.5-6)	0.767
	TC	23 (19-27)		5 (3-6.3)	
	CC	24.5 (19.3-30.8)		5 (4-7)	
	TC+CC	23 (19-28)	0.223	5 (4-7)	0.775
OCDS	TT	18 (6-24.5)	0.836	3 (2-4)	0.412
	TC	18 (12-27)		3 (2-5)	
	CC	16 (7.3-26)		3 (2-4)	
	TC+CC	17.5 (9.3-26.8)	0.688	3 (2-4)	0.822
Zung depression	TT	37 (29.5-47)	0.571	22 (20.5-22.5)	0.859
	TC	31 (26.3-44.5)		22 (20-25.3)	
	CC	35.5 (29-45.5)		22 (20-24.5)	
	TC+CC	33 (27-44.8)	0.513	22 (20-25)	0.584
Zung anxiety	TT	34 (28.5-46)	0.677	23 (21-25)	0.616
	TC	33 (28.3-38.5)		21.5 (20-23)	
	CC	34 (29-40.5)		21 (20-24.5)	
	TC+CC	33.5 (29-39)	0.723	21 (20-24)	0.360
BDHI	TT	36 (28-41.5)	0.607	17 (8-24.5)	0.995
	TC	32 (21.5-39.5)		15 (11-23)	
	CC	29 (22-40.8)		18 (10-23)	
	TC+CC	30 (22-40)	0.327	17 (11-23)	0.948

*Kruskal-Wall test for additive and Mann-Whitney test for dominant model

Supplementary Table S12. Associations between *IL1B* rs1071676 and the selected psychosymptomatology scale scores for hospitalized alcohol-dependent patients and healthy controls.

**Scale**	***IL1B* rs1071676 genotype**	**Patients (N=89)**		**Controls (N=93)**	
		Median (25-75%)	P*	Median (25-75%)	P*
YBOCS obsession	GG	1 (1-5.5)	0.712	1 (1-1)	0.375
	GC	2 (1-7)		1 (1-1.5)	
	CC	3.5 (1-15.8)		1 (1-4)	
	GC+CC	2 (1-7)	0.416	1 (1-2)	0.342
YBOCS compulsion	GG	1 (1-2.3)	0.573	1 (1-1)	0.092
	GC	1 (1-6)		1 (1-1)	
	CC	1 (1-2.5)		1 (1-2)	
	GC+CC	1 (1-5)	0.627	1 (1-1)	0.166
BSPS	GG	10 (3.8-17.3)	0.995	8 (5-12.5)	0.456
	GC	9 (4-19)		10 (6-16.5)	
	CC	11.5 (2-32.3)		8 (3-16)	
	GC+CC	9 (4-21)	0.923	10 (6-15)	0.431
AUDIT	GG	25 (20-30)	**0.045**	5 (4-6)	0.821
	GC	23 (19-27)		5 (3-7)	
	CC	13.5 (8.8-23.5)		4.5 (3.8-5.8)	
	GC+CC	22 (17-27)	0.069	5 (3-7)	0.770
OCDS	GG	17.5 (8.8-27.3)	0.360	3 (2-4)	0.950
	GC	20 (12-26)		3 (2-4)	
	CC	8 (3-25.8)		3 (2-4.5)	
	GC+CC	19 (9-26)	0.804	3 (2-4)	0.907
Zung depression	GG	32.5 (27.8-45)	0.751	22 (20-23.5)	0.885
	GC	35 (27-45)		22 (20.5-25.5)	
	CC	31 (24.3-46.8)		22 (20-27)	
	GC+CC	35 (27-45)	0.626	22 (20-26)	0.665
Zung anxiety	GG	32 (28-37)	0.054	22 (20-23.5)	0.868
	GC	36 (32-42)		21 (20-25.5)	
	CC	29.5 (23.8-45.8)		22.5 (20-24.3)	
	GC+CC	35 (31-42)	0.050	22 (20-25)	0.662
BDHI	GG	34.5 (21.8-42)	0.438	16 (11-25.5)	0.287
	GC	30 (25-37)		15 (9-20.5)	
	CC	24 (12-36)		21.5 (13.3-28)	
	GC+CC	30 (24-37)	0.380	18 (10-22)	0.664

*Kruskal-Wall test for additive and Mann-Whitney test for dominant model

Supplementary Table S13. Associations between *MIRN146A* rs2910164 and the selected psychosymptomatology scale scores for hospitalized alcohol-dependent patients and healthy controls.

**Scale**	***MIRN146A* rs2910164 genotype**	**Patients (N=89)**		**Controls (N=93)**	
		Median (25-75%)	P*	Median (25-75%)	P*
YBOCS obsession	GG	2 (1-7)	0.482	1 (1-2)	0.426
	GC	1 (1-4)		1 (1-1)	
	CC	2 (1-6)		1 (1-1)	
	GC+CC	1 (1-4.5)	0.236	1 (1-1)	0.478
YBOCS compulsion	GG	1 (1-3)	0.357	1 (1-1)	0.462
	GC	1 (1-3)		1 (1-1)	
	CC	2 (1-7)		1 (1-1)	
	GC+CC	1 (1-6)	0.580	1 (1-1)	0.315
BSPS	GG	10 (4-19)	0.152	8 (5-14)	0.680
	GC	7 (3-17)		10 (7-14)	
	CC	19 (5-26)		12 (4.5-15)	
	GC+CC	9.5 (3.8-18.3)	0.826	10 (7-14)	0.382
AUDIT	GG	25 (19-30)	0.576	5 (4-7)	0.218
	GC	22 (20-27)		5 (4-6)	
	CC	25 (23-29)		3 (2-5.5)	
	GC+CC	23 (20-28)	0.705	5 (3-6)	0.518
OCDS	GG	18 (9-28)	0.142	3 (2-4)	0.782
	GC	16 (7-23)		3 (2-4)	
	CC	22 (19-30)		2 (2-5)	
	GC+CC	18 (10.3-23.8)	0.891	3 (2-4)	0.626
Zung depression	GG	35 (29-44)	0.069	22 (20-24.5)	0.890
	GC	29 (25-40)		22 (20-24)	
	CC	45 (34-49)		22 (21-22.5)	
	GC+CC	30.5 (25-45.8)	0.222	22 (20-23.8)	0.630
Zung anxiety	GG	34 (29-39)	0.846	22 (21-24.5)	0.474
	GC	33 (29-40)		22 (20-24)	
	CC	35 (27-44)		21 (20.5-21.5)	
	GC+CC	33.5 (28.8-40.3)	0.842	22 (20-23.8)	0.402
BDHI	GG	32 (22-40)	0.823	18 (11.5-23.5)	0.178
	GC	30 (23-40)		15 (10-25)	
	CC	35 (14-46)		11 (6-15)	
	GC+CC	30 (22.8-40.3)	0.983	14.5 (10-21.8)	0.339

*Kruskal-Wall test for additive and Mann-Whitney test for dominant model

Supplementary Table S14. Associations between *IL6* rs1800795 and the selected psychosymptomatology scale scores for hospitalized alcohol-dependent patients and healthy controls.

Scale	*IL6* rs1800795 genotype	Patients (N=89)		Controls (N=93)	
		Median (25-75%)	P*	Median (25-75%)	P*
YBOCS obsession	GG	2 (1-7)	0.351	1 (1-1)	0.161
	GC	1 (1-5)		1 (1-2)	
	CC	1 (1-14)		1 (1-1)	
	GC+CC	1 (1-6)	0.259	1 (1-2)	0.190
YBOCS compulsion	GG	1 (1-5.5)	0.319	1 (1-1)	0.460
	GC	1 (1-2)		1 (1-1)	
	CC	2 (1-9.5)		1 (1-1)	
	GC+CC	1 (1-2)	0.487	1 (1-1)	0.483
BSPS	GG	9 (3-17.5)	0.684	7 (5-13)	0.590
	GC	10 (4-19)		9 (6.5-15.5)	
	CC	11 (5.5-28.5)		10 (6-14.5)	
	GC+CC	10 (4-19)	0.815	9 (6-14.5)	0.305
AUDIT	GG	23 (19.5-28.5)	0.646	4 (3-5)	0.093
	GC	24 (19-31)		5 (4-7)	
	CC	22 (16.5-28)		5 (2.5-6)	
	GC+CC	23 (19-28.8)	0.828	5 (4-7)	0.057
OCDS	GG	18 (8-28)	0.736	3 (2-4)	0.200
	GC	15 (8-26)		3 (2.5-4)	
	CC	17 (16-24.5)		3 (2-4)	
	GC+CC	16.5 (9.3-25.5)	0.543	3 (2-4)	0.187
Zung depression	GG	35 (28-46.5)	0.487	22 (21-24)	0.951
	GC	33 (27-45)		22 (20-25)	
	CC	32 (24.5-37)		22 (20-23.8)	
	GC+CC	32 (26.3-42.3)	0.382	22 (20-24.5)	0.923
Zung anxiety	GG	34 (29-39)	0.142	22 (21-25)	0.506
	GC	34 (30-40)		21 (20-24)	
	CC	29 (24.5-35.5)		21.5 (20-23)	
	GC+CC	33 (29-39)	0.705	21 (20-24)	0.260
BDHI	GG	30 (23.5-39)	0.783	17 (10-23)	0.413
	GC	32 (22-41)		14 (9-22)	
	CC	25 (21-39.5)		18.5 (12-28)	
	GC+CC	31 (22-40.8)	0.832	17 (10.5-22.5)	0.990

*Kruskal-Wall test for additive and Mann-Whitney test for dominant model

Supplementary Table S15. Associations between *IL6R* rs2228145 and the selected psychosymptomatology scale scores for hospitalized alcohol-dependent patients and healthy controls.

**Scale**	***IL6R* rs2228145 genotype**	**Patients (N=89)**		**Controls (N=93)**	
		Median (25-75%)	P*	Median (25-75%)	P*
YBOCS obsession	AA	2 (1-6)	0.123	1 (1-2)	0.510
	AC	1 (1-6.8)		1 (1-1)	
	CC	5 (1-12)		1 (1-1)	
	AC+CC	2 (1-9)	0.467	1 (1-1)	0.439
YBOCS compulsion	AA	1.5 (1-5.3)	**0.033**	1 (1-1)	0.127
	AC	1 (1-2)		1 (1-1)	
	CC	2 (1-9)		1 (1-1)	
	AC+CC	1 (1-3)	0.328	1 (1-1)	0.204
BSPS	AA	11 (4.8-23.3)	0.099	9 (7-17)	0.080
	AC	10 (4.3-17)		9.5 (6-13.8)	
	CC	4 (1-16)		4 (3.3-10.8)	
	AC+CC	9 (3-17)	0.104	8.5 (4-13)	0.313
AUDIT	AA	23 (19-29.3)	0.450	5 (4-8)	0.232
	AC	25 (20-28.8)		4.5 (3-6)	
	CC	22 (16-27)		5 (3.3-5)	
	AC+CC	24 (19-28)	0.749	5 (3-6)	0.088
OCDS	AA	18 (12-27.3)	0.808	3 (2-5)	0.298
	AC	18 (8-26)		3 (2-4)	
	CC	16 (7-27)		3 (3-4)	
	AC+CC	18 (8-26)	0.542	3 (2-4)	0.269
Zung depression	AA	33.5 (27-48)	0.186	22 (21-25)	0.173
	AC	35.5 (29-44.8)		22 (20.3-24)	
	CC	32 (23-37)		21 (20-22.8)	
	AC+CC	34 (27-42)	0.517	21.5 (20-23.8)	0.096
Zung anxiety	AA	32.5 (27.8-41.3)	0.467	22 (21-25)	0.440
	AC	34.5 (30.5-38.8)		22 (20.3-24)	
	CC	31 (29-36)		21 (20-22.8)	
	AC+CC	34 (30-38)	0.558	21.5 (20-24)	0.465
BDHI	AA	29.5 (19.8-40.3)	0.739	20 (14-26)	**0.014**
	AC	33 (27.3-38)		14.5 (8.5-22.5)	
	CC	27 (21-41)		12 (11-16.8)	
	AC+CC	32 (25-40)	0.595	14 (10-20.8)	**0.004**

*Kruskal-Wall test for additive and Mann-Whitney test for dominant model

Supplementary Table S16. Thermal cycling conditions used for *GPX1* rs1050450, *IL1β* rs1143623 and rs16944, *IL6* rs1800795, *IL6R* rs2228145, miR146a rs2910164, *PON1* rs854560 and rs662, and *SOD2* rs4880 genotyping.

	**61-55°C Touchdown protocol**		
**Stage**	**Temperature**	**Duration**	**Number of cycles**
Hot-start Taq activation	94°C	15 min	1
Touchdown	94°C	20 s	10
	61°C (61°C decreasing 0.6°C per cycle to achieve a final annealing / extension temperature of 55°C)	60 s	
Amplification	94°C	20 s	30
	55°C	60 s	
Read stage	30°C	60 s	1

Supplementary Table S17. Thermal cycling conditions used for *PON1* rs705379 and rs705381 genotyping.

	**68-62°C Touchdown protocol**		
**Stage**	**Temperature**	**Duration**	**Number of cycles**
Hot-start Taq activation	94°C	15 min	1
Touchdown	94°C	20 s	10
	68°C (68°C decreasing 0.6°C per cycle to achieve a final annealing / extension temperature of 62°C)	60 s	
Amplification	94°C	20 s	30
	62°C	60 s	
Read stage	30°C	60 s	1

Supplementary Table S18. Thermal cycling conditions used for *IL1β* rs1071676 genotyping.

	**68-62°C Touchdown protocol**		
**Stage**	**Temperature**	**Duration**	**Number of cycles**
Hot-start Taq activation	94°C	15 min	1
Amplification	94°C	20 s	36
	57°C	60 s	
